# Role of primary chemoradiotherapy in the management of advanced stage vulvar cancer: Experience of a large cancer center

**DOI:** 10.1007/s00066-025-02483-z

**Published:** 2025-11-10

**Authors:** Lars Wessel, Maria Vinsensia, Thomas Koenigsmann, Juergen Debus, Nathalie Arians

**Affiliations:** 1 Department of Radiation Oncology, Heidelberg University Hospital, Heidelberg, Germany; 2 Heidelberg Institute of Radiation Oncology (HIRO), Heidelberg, Germany; 3 National Center for Tumor diseases (NCT), Heidelberg, Germany; 4 Department of Radiation Oncology, Municipal Hospital Ludwigshafen, Ludwigshafen, Germany; 5 Clinical Cooperation Unit Radiation Oncology, German Cancer Research Center (DKFZ), Heidelberg, Germany; 6 Heidelberg Ion-Beam Therapy Center (HIT), Department of Radiation Oncology, Heidelberg University Hospital, Heidelberg, Germany; 7 German Cancer Consortium (DKTK), Partner Site Heidelberg, Heidelberg, Germany

**Keywords:** Lymph nodes, Definitive radiotherapy, Concurrent chemotherapy, Treatment outcome, Potential predictive factors

## Abstract

**Purpose:**

Treatment of advanced vulvar cancer is challenging. The aim of the study was to investigate the potential of primary (chemo)radiotherapy ((C)RT) with regard to clinical outcome and associated prognostic parameters.

**Methods:**

A total of 39 patients with squamous cell vulvar cancer receiving primary (C)RT were retrospectively identified through hospital databases. Patient and treatment characteristics as well as outcomes were assessed. Survival statistics were calculated using the Kaplan–Meier method. Univariate analysis was performed using the log-rank test and Spearman’s correlation to evaluate associations between patient or treatment characteristics and survival outcomes.

**Results:**

Median age at diagnosis was 74 years (range 38–92 years). Patients had advanced stage disease with 28.2%/38.5% presenting with FIGO stage III/IV, respectively. All patients received external beam radiotherapy (EBRT) with a median dose to the primary tumor of 66 Gy EQD2 (range 49.6 Gy–72.6 Gy) and to lymph nodes of 53.1 Gy EQD2 (range 44.1 Gy–67.1 Gy). 69.2% received concurrent chemotherapy, mostly cisplatin weekly or mitomycin/5-fluorouracil. 10.3%/64.1% showed clinical complete remission (cCR)/partial remission (cPR) at first follow-up; 7.7% had disease progression. After a median follow-up of 25.5 months (range 0.5–132.9 months), 3‑year locoregional progression-free survival (LRPFS) and overall survival (OS) were 60.2% and 69.6%, respectively. Age and concurrent chemotherapy were the main prognostic parameters associated with improved oncological outcome.

**Conclusion:**

Definitive (C)RT plays an important role in the management of advanced vulvar cancer with high response rates and satisfactory oncological outcomes. However, there is still room for improvement and future trials are needed to further assess the potential of definitive CRT, especially with regard to possible combinations with immunotherapy.

## Introduction

Vulvar cancer is a rare disease with an age standardized rate of about 0.9/100,000 women worldwide in 2020 (about 45,000 cases worldwide) [1]. Regions with the highest incidence rates are Western and Northern Europe, Northern America, Australia, and New Zealand followed by Eastern Africa [2]. Vulvar cancer usually affects older women with the mean age at diagnosis of 73 years in Germany [3]. However, numbers are increasing—especially the incidence of human papillomavirus-associated cancers is rising [4]. Primary therapy routinely consists of surgical approaches. Surgery aims at complete resection of the tumor with clear pathological margins. The minimal pathological margin distance is controversially discussed, some postulating a margin of at least 8 mm, while others showing no prognostic impact of pathological margin distance [5–7]. Pathological staging of inguinofemoral lymph nodes depends on tumor size, depth of infiltration, and location of the tumor. Depending on risk factors (e.g., no clear resection margins, extensive lymphovascular space involvement, tumor size or pathological lymph node involvement), postoperative radiotherapy (RT) or even chemoradiotherapy (CRT) of the vulva and/or lymphatic drainage may be indicated. According to the literature, 5‑year overall survival (OS) and disease-free survival rates over all patients are 61.4% and 48.1%, respectively [8]. But numbers are very much dependent on tumor stage. According to the Robert Koch Institute (RKI), relative 5‑year OS of vulvar cancer patients in Germany ranges from 88% in UICC stage I disease to 19% in stage IV disease [3].

Treatment of advanced vulvar cancer is challenging. Extensive tumors with close proximity to the clitoris, urethra, or anus may not be suitable for standard surgical approaches. Pelvic exenteration with high rates of perioperative mortality and morbidity is the only surgical option with a negative impact on quality of life [9–11]. Other reasons for inoperability may be patients’ comorbidities rendering them functionally inoperable or recurrent disease after prior extensive surgery. Preoperative or primary definitive RT or CRT are alternative options to extensive surgery. Randomized data of the comparison of extensive surgery and primary (C)RT or neoadjuvant (C)RT are lacking. However, there are several retrospective reports and a few prospective but nonrandomized trials addressing this issue.

A retrospective analysis from Landrum et al. [12] tried to compare outcome measures in patients with advanced squamous cell carcinoma of the vulva treated with surgery or primary CRT. A total of 63 patients with stage III and IV were included in this analysis: 30 patients were treated with surgery, and 33 patients had tumors that were unresectable by vulvectomy and thus received primary CRT. There were no statistically significant differences between the two groups with OS rates of 69% (surgery) vs 76% (CRT). In multivariate analysis, age was the only predictor for worse OS and PFS.

A National Cancer Database analysis [13] tried to assess primary versus preoperative radiation followed by surgery for locally advanced vulvar cancer. Included were 2046 women who were treated for vulvar cancer from 2004–2012. Primary RT/CRT was associated with compromised 3‑year OS, compared with preoperative RT/CRT + surgery (41.7% vs 57.1%, respectively; *p* = 0.001). On multivariate analysis, OS associated with primary RT/CRT with doses more than 55 Gy was not significantly different from RT/CRT + surgery. Use of concurrent chemotherapy improved OS in both groups.

There are two prospective phase II trials evaluating definitive CRT for locally advanced squamous cell cancer of the vulva. One of them [14] treated 52 patients with mainly T2 and T3 disease from 2007 to 2019 with radiotherapy and capecitabine. In all, 62% showed clinical complete response 12 weeks after treatment. The 2‑/5-year PFS was 51%/45% and OS was 66%/52%. In the other prospective study [15], 58 patients with advanced vulvar cancer (T3/T4 tumors not suitable for radical vulvectomy) received radiotherapy with concurrent cisplatin 40 mg/m^2^ body surface area weekly. Overall, 64% showed complete clinical response and 78% even pathological response confirmed by biopsy 6–8 weeks after CRT.

All these trials could show that definitive CRT plays an important role in the management of advanced and unresectable vulvar cancer with high response rates and satisfactory oncological outcomes, yet many questions remain unanswered. For optimal clinical management of this patient collective and to further improve oncological outcomes much more information is needed, for example, about the correct patient selection, radiotherapy dosage, radiotherapy targets or chemotherapy regimens. To further answer these questions, we performed a retrospective analysis of all vulvar cancer patients who received primary radiotherapy at one institution. Aim of the study was to investigate clinical features and outcomes of patients with vulvar cancer who underwent primary radiotherapy with regard to clinical outcome and potential predictive factors.

## Patients and methods

For this analysis, we reviewed all patients with squamous cell carcinoma of the vulva who have been treated with radiotherapy at Heidelberg University Hospital between 05/2005 and 09/2024 using the clinical cancer registry of the National Center for Tumor Diseases (NCT). A total of 39 patients who received primary radiotherapy in a potentially curative setting were identified. Each patient was discussed in an interdisciplinary tumor board and indication for primary radiotherapy was confirmed. A computerized database was used to review the medical records in order to abstract patient and treatment characteristics.

The following clinical data were collected: age, histology, grading, tumor stage including lymph node status according to the TNM and FIGO classification of 2021, date of first diagnosis, performance of radiotherapy including dose, radiation field, use of sequential or simultaneous boost to the primary tumor and/or lymph node metastases, time to recurrence, pattern of recurrence like local recurrence of the vulva and/or lymph node recurrence, onset and location of distant metastases, and date of death.

Overall survival (OS), local progression-free survival (LPFS), regional progression-free survival (RPFS), locoregional progression-free survival (LRPFS), and distant progression-free survival (DPFS) were evaluated. Statistical events were defined as death from any cause (OS), local relapse at the vulva (LPFS), relapse at inguinal lymph nodes (RPFS), local relapse at the vulva or inguinal lymph nodes (LRPFS), and occurrence of any distant metastases (DPFS). Time to event data was measured from the date of last radiotherapy treatment to the date of recurrence or the date of last follow-up with information about the oncological status. Overall survival was equally measured from the date of last radiotherapy treatment until death from any cause or last follow-up, using the NCT cancer registry with a deadline of 6 May 2025.

Statistical analysis was performed using SPSS (version 29; IBM Corp., Armonk, NY, USA) and GraphPad Prism version 10.5. (GraphPad Software, Boston, MA, USA, www.graphpad.com). Survival statistics were calculated using the Kaplan–Meier method. Univariate analysis for comparison of subgroups was performed using the log-rank test. Spearman’s correlation was employed to evaluate associations between patient or treatment characteristics and survival outcomes. A *p* value < 0.05 was considered statistically significant.

All data were collected retrospectively and in accordance with institutional ethical policies. The study was granted ethical approval by the local ethics committee of Heidelberg University (S-808/2019).

## Results

Between May 2005 and September 2024, 39 patients were identified who received primary radiotherapy in a potentially curative setting. Of them, 6 were planned to receive preoperative chemoradiotherapy followed by surgical resection, but the concept was changed during the course of treatment and these patients also received definitive chemoradiotherapy. Furthermore, 8 patients had prior history of vulvar carcinoma and received primary radiotherapy at time of recurrence, while 31 patients were treated in a primary setting at time of first diagnosis.

Patient characteristics are summarized in Table 1. Median age at diagnosis was 74 years (range 38–92 years). All patients had squamous cell carcinoma of the vulva, 71.8% keratinizing and 20.5% nonkeratinizing subtypes. All patients were staged by gynecological examination, 23 patients underwent pelvic MRI and CT imaging, and 16 patients underwent CT only and were classified using the FIGO classification of 2021. One patient presented with FIGO IB disease, 12 patients (30.8%) were classified FIGO stage II, 11 patients (28.2%) FIGO stage III, and 15 patients (38.5%) FIGO stage IV, respectively. Four patients (10.3%) had G1, 27 (69%) G2, and 8 (20.5%) G3 tumors. 15 patients (38.5%) had negative lymph nodes at diagnosis. 14 patients (35.9%) presented with inguinal lymph node metastases only, 4 (10.3%) patients had inguinal and pelvic lymph node metastases, 3 patients (7.7%) had pelvic lymph nodes only, and 3 patients (7.7%) had inguinal, pelvic and para-aortic lymph node metastases.

**Table 1:** Patient demographics and tumor characteristics. Median and range are specified where applicable; number of cases and percentage are stated

	Median (range)	Cases (*n*)	%
*Age at diagnosis*	74 years (38–92)	–	–
*FIGO stage (2021)*			
IB	–	1	2.6
II		12	30.8
IIIA		4	10.3
IIIB		7	17.9
IVA		6	15.4
IVB		9	23.1
*Grading*			
G1	–	4	10.3
G2		27	69.2
G3		8	20.5
*Histology*			
Nonkeratinizing SCC	–	8	20.5
Keratinizing SCC		28	71.8
SCC, no further information		3	7.7
*Lymph nodes involved*			
No	–	15	38.5
Inguinal		21	53.8
Pelvic		10	25.6
Para-aortal		3	7.7
*EBRT field*			
Vulva	–	4	10.3
Vulva + inguinal		18	46.1
Vulva + inguinal + pelvic		13	33.3
Vulva + inguinal + pelvic + para-aortal		4	10.3
*Concurrent chemotherapy*			
Yes	–	27	69.2
No		12	30.8
*Overall treatment time*	43 days (23–64)	**–**	**–**

*SCC* squamous cell carcinoma, *EBRT* external beam radiotherapy

All patients received external beam radiotherapy (EBRT) in a curative setting. Median treatment time was 43 days (range 23–64 days). All patients received a cumulative dose to the vulva of at least 50 Gy EQD2: 35 patients received a cumulative dose to the vulva > 55 Gy EQD2, 30 patients > 60 Gy EQD2, and 26 patients > 65 Gy EQD2. Doses to the lymph nodes were generally lower than doses to the vulva: 16 patients received a cumulative dose to the lymph nodes > 55 Gy EQD2, 10 patients > 60 Gy EQD2, and only 8 patients > 65 Gy EQD2. Dose characteristics are displayed in detail in Table 2. The radiation field consisted of the vulva only in 4 patients (10.3%), the vulva and bilateral inguinal lymphatic drainage in 18 patients (46.2%), the vulva and bilateral inguinal and pelvic lymphatic drainage in 13 patients (33.3%), and 4 patients (10.3%) received additional radiation to the para-aortic nodal region. All patients who received radiotherapy to the vulva only were staged cN0. Nine (50%) of the 18 patients who received radiotherapy to the vulva and inguinal nodal region were staged cN0; 9 patients (50%) had suspicious inguinal lymph nodes. Only 5 of the 13 patients (38.5%) who received additional pelvic radiation had pelvic lymph node metastases; the others had inguinal lymph node metastases only (7 patients; 53.8%) or no lymph node metastases at all (1 patient; 7.7%). All 3 patients who had para-aortic lymph node metastases received additional para-aortic radiation; 1 patient with only inguinal and pelvic lymph node metastases also received additional para-aortic radiation.

**Table 2:** Dose characteristics of EBRT

	*n*	Min	Max	Median	Mean
*Elective dose (EQD2)*	–	4.1	56	50	50.3
*Boost vulva*	37	–	–	–	–
SIB	17				
Sequential	29				
Brachytherapy	1				
*Dose of boost to the vulva (EQD2)*	–	5.5	71.6	17.7	38.5
*Cum. dose to the vulva (EQD2)*	–	49.6	72.6	66	62.2
*Boost lymph nodes*	17	–	–	–	–
SIB	10				
Sequential	7				
*Dose of boost to the lymph nodes (EQD2)*	–	8.9	67.1	58.1	45.9
*Cum. dose to the lymph nodes (EQD2)*	–	44.1	67.1	53.1	53.1

*Cum.* cumulative; *EBRT* external beam radiotherapy; *EQD2* Equivalent dose in 2 Gy fractions; *Max* Maximum; *Min* Minimum; *SIB* simultaneous integrated boost

In all, 27 patients (69.2%) received concurrent chemotherapy, mostly cisplatin weekly (13 patients; 48.2%) or mitomycin/5-fluorouracil (FU; 12 patients; 44.4%). One patient (3.7%) received carboplatin AUC2 and 1 patient (3.7%) gemcitabine.

Median time of follow-up was 25.5 months (range 0.5–132.9 months). A total of 36 patients received radio-oncological follow-up visits; 3 patients died shortly after finishing treatment and before the first follow-up visit. 11 patients received CT and 23 MRI at first follow-up, 2 patients had clinical follow-up examination without imaging or gynecological examination, so that remission status at first follow-up was unclear. Four patients showed complete remission and 25 patients partial remission at first follow-up; 2 showed at least stable disease. Three patients showed progressive disease at first follow-up with progression of lymph node metastases (2 patients) and newly diagnosed lymph node metastases (1 patient). One of them also developed local recurrence in the vulva later in the course of follow-up. Two of them also developed distant metastases in the lung and bone. All of them died (2.6, 5.8, and 16.1 months) after end of radiotherapy.

Ten patients developed local recurrence of the vulva, 5 together with lymph node recurrence, 2 developed lymph node recurrence in the further course of disease, and 1 had lymph node recurrence before local recurrence. All in all, 10 patients developed lymph node recurrences, 3 of these patients were initially staged cN0 and 3 of them had isolated lymph node recurrences. Local recurrences occurred after a median time of 10.6 months. Lymph node recurrences occurred after a median time of 11.6 months. Four patients (10.3%) developed distant metastases in the course of follow-up after a median time of 11.6 months. Three of them also had locoregional recurrence, while 1 had isolated distant recurrence in the lung and lymph nodes outside the pelvis.

During the course of follow-up, 18 patients (46.2%) died. Oncological outcome parameters of the cohort are listed in Table 3.

**Table 3:** Oncological outcome of the whole cohort. Numbers of cases and percentage are stated

Oncological outcome	Cases (*n*)	%
*Local recurrence*		
Yes	10	25.6
No	26	66.7
Unknown	3	7.7
*Lymph node recurrence*		
Yes	10	25.6
No	26	66.7
Unknown	3	7.7
*Distant metastases*		
Yes	4	10.3
No	32	82
Unknown	3	7.7
*Death*		
Yes	18	46.2
No	21	53.8
*Response at first follow-up*		
CR	4	10.3
PR	25	64.1
SD	2	5.1
PD	3	7.7
Unknown	5	12.8

*CR* complete remission; *PD* progressive disease; *PR* partial remission; *SD* stable disease

**Fig. 1:**
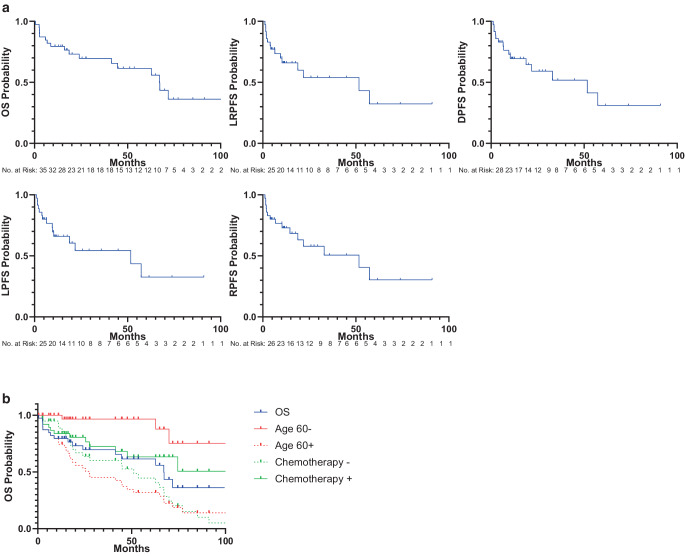
**a** Kaplan–Meier-estimated overall survival (OS), local progression-free survival (LPFS), regional progression-free survival (RPFS), locoregional progression-free survival (LRPFS), and distant progression-free survival (DPFS) and **b** results of the log-rank test showing the most important parameters

Kaplan–Meier-estimated median OS was 67.15 months with 1‑/3- and 5‑year OS rates of 79.5%/69.6% and 61.4%, respectively. Kaplan–Meier-estimated median LRPFS was 51.7 months with 1‑/3- and 5‑year LRPFS rates of 66.3%/60.2% and 36.1%, respectively. Kaplan–Meier-estimated median LPFS was 57.3 months with 1‑/3- and 5‑year LPFS rates of 73.7%/67.5% and 40.5%, respectively. Kaplan–Meier-estimated median RPFS was 57.3 months with 1‑/3- and 5‑year RPFS rates of 80.4%/62.6% and 46.9%, respectively. The 1‑/3- and 5‑year DPFS rates were 90.1%/90.1% and 72.1%, respectively. Kaplan–Meier curves are shown in Fig. 1a.

**Fig. 2:**
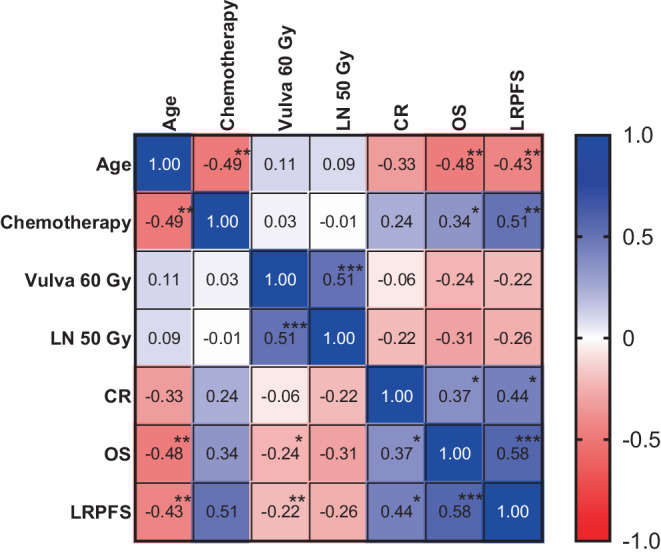
Results of representative parameters tested with Spearman’s correlation. *p* < 0.05*; *p* < 0.01**; *p* < 0.001***; *CR* complete remission; *EQD2* equivalent dose in 2 Gy fractions; *LN* lymph nodes; *LRPFS* locoregional progression-free survival; *OS* overall survival; *sim.* simultaneous

Spearman’s test was performed to identify potential prognostic parameters. Age at first diagnosis, the use of simultaneous chemotherapy and clinical complete remission at first follow-up correlated significantly with all oncological outcome parameters like OS, LRPFS, LPFS, RPFS, and DPFS. Details including *p*-values and Spearman coefficient are displayed in Fig. 2.

Survival time comparisons of different group partitions were performed using log-rank test. Results including *p*-values are displayed in Table 4. The use of concomitant chemotherapy was a significant prognostic factor for improved LPFS, LRPFS, and a trend for a better OS and RPFS (Fig. 1b). The tumor response assessed at first follow-up was also a significant prognostic factor for OS, LPFS, RPFS, LRPFS, and DPFS with patients having CR or PR showing improved outcome. The occurrence of distant metastases was prognostic for OS, RPFS, and LRPFS, the radiation field was prognostic for OS and RPFS. Age was a strong prognostic factor with patients aged 60 years or older showing significantly worse OS and LRPFS (Fig. 1b) and patients aged 70 years or older showing significantly worse OS and LPFS. Age had no impact on RPFS and DPFS. Survival time comparisons of group partitions with different dose cut-off values were performed using log-rank test. No statistically significant dose parameters or dose cut-offs relevant for oncological outcome could be identified.

**Table 4:** Results of univariate analysis using log-rank test

	Mean OS	*p*-value	Mean LPFS	*p*-value	Mean RPFS	*p*-value	Mean LRPFS	*p*-value	Mean DPFS	*p*-value
*Chemotherapy*	–	0.051	–	0.01*	–	0.068	–	0.004*	–	0.588
Yes (27)	79.9		59.7		63.1		7.9		76.2	
No (12)	34.2		9.8		20		56.5		29	
*Response at first FU*	–	< 0.001*	NA	0.054	NA	< 0.001*	NA	< 0.001	NA	< 0.001
CR	74.4									
PR	81.4									
SD	67.2									
PD	8.2									
NA	27.1									
*RT field*	–	< 0.001*	–	0.474	–	0.01*	–	0.135	–	–
vulva	21.3		8.6		27.5		8.6			
vulva + ing.	53.9		57.5		56.6		48.5			
vulva + ing. + pel.	92.4		56		61.5		56.3			
vulva + ing. + pel. + para-aortic	7.1		10.3		6.2		6.2			
*Age* *≥* *60*	–	0.028*	–	0.065	–	0.102	–	0.034*	–	–
Yes	56.7		38.3		37.7		33.2			
No	85.6		76.5		76.5		76.5			
*Age* *≥* *70*	–	0.008*	–	0.027*	–	0.112	–	0.06	–	0.411
Yes	40.9		34.5		33.4		30.9		54.3	
No	106.5		69.9		71.6		64.1		70.5	
*Age* *≥* *80*	–	0.002*	–	0.287	–	–	0.657	0.296	–	–
Yes	22.7		13.1			24.5				
No	83.9		56.6			57				
*Local recurrence*	–	< 0.001*	–	–	–	0.011*	–	–	–	0.483
Yes	48.6				28.4				48.5	
No	72				79				83.4	
*Regional recurrence*	–	< 0.001*	–	< 0.001*	–	–	–	–	–	0.31
Yes	62.8		21.7						45.3	
No	132.9		73.5						77.8	
*Distant metastases*	–	< 0.001*	–	0.172	–	0.042*	–	0.009*	–	–
Yes	38.5		30.7		2		18.5			
No	64.6		59.8		57.3		54.9			

*CR* complete remission; *FU* Follow-up; *ing.* inguinal; *NA* not assessed; *PD* progressive disease; *pel.* pelvic; *PR* partial remission; *SD* stable disease* LPFS* local progression-free survival; *LRPFS* locoregional progression-free survival; *RPFS* regional progression-free survival; *DPFS* distant progression-free survival; *OS* overall survival, *RT* radiotherapy

* statistically significant

## Discussion

Vulvar cancer is a rare disease and only few prospective data regarding the role of primary radiotherapy exist. Some retrospective analyses with more or less small patient cohorts tried to address this issue, including the need of simultaneous chemotherapy, the correct target volume definition, or radiotherapy dosage.

Our collective with 39 patients is a rather large cohort regarding the low incidence rates and the fact that most patients are diagnosed in earlier stages and are eligible for surgical resection. The negative patient selection in our study has to be kept in mind when interpreting the results. 28.2% presented with FIGO stage III and 38.5% with stage IV disease. Some patients already had pelvic or even para-aortal lymph node metastases at time of first diagnosis. Eight patients had prior history of vulvar cancer and were treated for recurrent disease. All patients were assumed inoperable by standard surgical approaches and primary radiotherapy was the recommended treatment, in a potentially curative setting. The 5‑year OS in our cohort was rather good with 61.4%; 25.6% of the patients developed local or lymph node recurrences with a median LRPFS of 51.7 months and a 3-year LRPFS rate of 60.2%. Distant metastases seem to play a minor role for progression-free survival in vulvar cancer patients; in our cohort 5-year DPFS was 72.1%. Similar results were reported by the retrospective KROG 1203 study [16]. This trial also included mainly patients with advanced stage disease (*n* = 56) with 36% stage III and IV, respectively. After a median follow-up of 2.8 years, 21 patients (37.5%) experienced treatment failure: 15 patients (27%) had local failure, and 7 (13%) even had persistent disease at the first follow-up. The 5‑year OS and DFS were 51.6% and 44.0%, respectively. OS in this cohort is worse than in our cohort. This might be due to the fact that only 41% received concurrent CRT compared to 69.2% in our cohort. The application of concurrent chemotherapy was a statistically significant prognostic parameter for oncological outcome in our analysis, which has already been reported in literature [13, 17, 18]. Patients receiving concurrent chemotherapy showed a mean OS of 80 months compared to 34 months for patients receiving radiotherapy only. In the NCDB analysis from Rao et al. [17], the use of concurrent chemotherapy improved OS significantly with 5‑year OS rates of 49.9% vs 27.4% without chemotherapy. Multivariate analysis even showed a reduced hazard of death by the use of concurrent chemotherapy. In the prospective phase II study of definitive CRT from van Triest et al. [14], 5‑year PFS and OS rates were 45% and 52%, respectively. Local clinical complete response (cCR) and regional control (RC) rates assessed 12 weeks after completing therapy were 62% and 75%, respectively. After 2 years, local cCR persisted in 22 patients (42%) and RC was 58%. 58% of patients had no evidence of disease at the end of follow-up (median 35 months). This trial mostly included patients with T2 and T3 tumors, all patients received chemoradiotherapy with capecitabine and a tumor dose of 64.8 Gy. In our cohort, median tumor dose was similar with 66 Gy EQD2, but only 69.2% received concurrent chemotherapy, either with cisplatin weekly or with mitomycin/5-FU. Nevertheless, OS was better in our cohort. Another prospective phase II trial evaluating definitive RT with cisplatin weekly [15] performed biopsies 6–8 weeks after finishing CRT to assess response with 37 patients showing complete clinical response (64%) and 29 patients (78%) even showing complete pathological response. One weakness of our trial is that patients received no biopsy after end of treatment to assess pathological response. Even the clinical follow-up examinations were not standardized, which is a result of the retrospective nature of our study. Response rates were retrospectively assessed by extracting the CT or MRI reports from the clinical database; only few patients had additional information about gynecological examinations at our institution. This is why only few patients were classified as CR at first follow-up; most patients had PR because radiological reports were not unequivocally negative. However, response at first follow-up was a statistically significant parameter in our cohort with a mean OS of 81.4 months and 67.2 months for patients showing CR or PR, respectively. But due to the small number of patients classified as CR, these data have to be interpreted with caution.

Patterns of care for vulvar cancer patients differ a lot between institutions, which is a large challenge for defining the optimal treatment regimen and also for interpretation of reported data. There are no prospective data nor is there a clear consensus on dosage for primary radiotherapy or target volume definition. A Gynecologic Cancer Intergroup (GCIG) study [19] tried to assess the ongoing practice of radiotherapy for the treatment of vulvar cancer in member study groups of the GCIG via a survey. Unfortunately, only neoadjuvant and adjuvant radiotherapy practices were assessed; there is no distinction between neoadjuvant and definitive radiotherapy. In this analysis, the main indication for neoadjuvant radiotherapy were unresectable disease or FIGO stage ≥ III. Only 81% used concomitant chemotherapy.

In our analysis, RT field was a statistically significant parameter for regional control with improved control rates for patients receiving inguinal + pelvic irradiation (mean RPFS 61.5 months for patients receiving irradiation to the vulva + inguinal + pelvic vs 56.6 months for patients receiving irradiation to the vulva + inguinal vs 27.5 months for patients receiving irradiation to the vulva only). However, these data have to be interpreted with caution. There is no common consensus on the target volume definition depending on tumor stage and even in one department there are large variations especially regarding the inclusion of the pelvic lymph node levels. In our study, 46.2% received irradiation of the vulva + inguinal lymphatic drainage, while in 43.6% of patients pelvic lymphatic drainage was also included. In the KROG study, for example, the majority (75%) received radiotherapy to the vulva + inguinal + pelvic lymphatic drainage [16].

Some of the retrospective trials assessing the role of primary RT for vulvar cancer reported dose parameters or even tried to assess the prognostic role of different dose parameters. Dose parameters from 2046 patients extracted in a NCDB analysis [13] showed a median dose to the vulva of 45 Gy (interquartile range [IQR] 42.2–50.4 Gy) with an additional median boost dose of 16 Gy (IQR 10–20 Gy) in 53%. The 3‑year OS rates were significantly better when applying doses > 55 Gy (50.3%) compared to doses ≤ 55 Gy (33%). In a retrospective analysis of 25 patients on definitive CRT, median dose to the vulva was 66 Gy (IQR 66–68 Gy), whereas median dose to lymph node metastases was 60.6 Gy (IQR 55.9–62.6 Gy) using simultaneous integrated boost concepts. Overall, 88% showed clinical complete remission; 2‑year OS was 63% [20]. In the prospective trial from Triest et al. [14], mean radiotherapy dose to the primary tumor was 64.1 Gy and mean elective dose to the nodal regions was 49.0 Gy (range 27.0–52.2 Gy) and 2 patients received a sequential boost on remaining lymph node metastases (61.2 and 64.8 Gy), showing a 5-year PFS of 45% and a 5-year OS of 52%. Another trial assessed primary RT for patients with gross inguinal lymphadenopathy. Patients received a median dose to the lymph nodes of 66 Gy (range 60–70 Gy), resulting in a 3-year OS of 51%. Rates of local recurrence of the vulva and inguinal after 3 years were 24.2% and 17.7%, respectively [21]. In our cohort, median dose to the vulva was 66 Gy (EQD2; range 49.6–72.6) resulting in a 3-year LPFS of 67.5%. Median dose to lymph nodes was 53.1 Gy (EQD2; range 44.1–67.1 Gy), resulting in a 3-year RPFS of 62.6%. Univariate analysis using different dose cut-offs did not show any significant correlation with any oncological outcome measure like LRPFS or OS. This might be due to the rather heterogenous use of dose and fractionation regimens, which have also changed during the time period of treatment of the included patients, and also due to the small patient collective with many different tumor stages.

In most of the studies, age is one of the strongest predictors for worse OS and even PFS [12, 16]. For example, in multivariate analysis reported in the KROG 1203 study, clinical size ≥ 3 cm and age ≥ 70 years were poor prognostic factors for DFS (*p* = 0.040 and *p* = 0.032, respectively) and age also for OS (*p* = 0.048). Our analysis also showed age to be a statistically significant prognostic parameter with patients aged 60 years or older showing significantly worse OS and LRPFS.

Neoadjuvant RT/CRT is usually applied for the treatment of other tumor entities in advanced stage disease or if patients are deemed borderline operable or inoperable. In the management of vulvar cancer, neoadjuvant RT/CRT can also be applied. However, there are no prospective data on the use of neoadjuvant RT/CRT; especially data comparing neoadjuvant and definitive RT/CRT are missing. A NCDB analysis [13] showed compromised OS rates for primary RT/CRT compared to RT/CRT followed by surgery. On multivariate analysis, OS did not differ any more between groups when applying doses more than 55 Gy for women receiving primary RT/CRT. In general, concurrent chemotherapy was associated with improved OS in both groups. A multivariate analysis of all patients receiving concurrent chemotherapy showed no difference in OS of women receiving primary CRT with doses > 55 Gy (3-year OS 56.9%) versus neoadjuvant CRT + surgery (3-year OS 58.3%). These data show the importance of concurrent chemotherapy and adequate dosing of radiotherapy for achieving long-term remission.

## Conclusion

Altogether, definitive chemoradiotherapy (CRT) is a valid treatment option for patients with advanced vulvar cancer with high response rates and oncological outcomes comparable to surgical approaches. The addition of chemotherapy plays a crucial role for a successful treatment, but there is still room to improve oncological outcome. Recent data reported about the addition of immunotherapy to definitive CRT. In a single arm phase II trial, 24 patients were treated with definitive CRT using cisplatin weekly and pembrolizumab, showing an overall response rate (complete remission+ partial remission) of 75% and a 6-month RFS (recurrence free survival) of 70% [22]. On the other hand, 78.6% experienced grade 3 or 4 toxicity, immune-related toxicities were mostly rated grade 1 or 2. All patients showed programmed death-ligand 1 (PD-L1) expression with a CPS (combined positive score) ≥ 1. Future trials are needed to further assess the potential of definitive CRT, especially with regard to possible combinations with immunotherapy, taking into account the fact that patients are mostly of older age and the risk for toxicity and thus impact on quality of life.
